# Identifying Predictors of Cervical Cancer Screening Uptake in Sub-Saharan Africa Using Machine Learning: Cross-Sectional Study

**DOI:** 10.2196/71677

**Published:** 2025-09-17

**Authors:** Nebebe Demis Baykemagn, Mekuriaw Nibret Aweke, Amare Mesfin, Lemlem Daniel Baffa, Muluken Chanie Agimas, Habtamu Wagnew Abuhay, Dagnew Getnet Adugna, Tewodros Getaneh Alemu, Alemu Teshale Bicha, Gebrie Getu Alemu

**Affiliations:** 1 Department of Health Informatics, Institute of Public Health, College of Medicine and Health Sciences, University of Gondar, Gondar, 196, Ethiopia, 251 918310345; 2 Department of Human Nutrition, Institute of Public Health, College of Medicine and Health Sciences, University of Gondar, Gondar, Ethiopia; 3 Department of Epidemiology and Biostatistics, Institute of Public Health, College of Medicine and Health Sciences, University of Gondar, Gondar, Ethiopia; 4 Department of Human Anatomy, School of Medicine, College of Medicine and Health Science, University of Gondar, Gondar, Ethiopia; 5 Department of Pediatrics and Child Health Nursing, School of Nursing, College of Medicine and Health Sciences, University of Gondar, Gondar, Ethiopia; 6 Department of Obstetrics and Gynecology, School of Medicine, College of Medicine and Health Sciences, University of Gondar, Gondar, Ethiopia

**Keywords:** cervical cancer, digital health, machine learning, woman, artificial intelligence

## Abstract

**Background:**

Cervical cancer has been ranked as the fourth most common cancer affecting women, contributing to approximately 660,000 new diagnoses and 350,000 fatalities worldwide. Effective early screening has been shown to reduce cervical cancer incidence by up to 80% and prevent more than 40% of new cases.

**Objective:**

This study aims to assess a machine learning–based prediction model and identify the key predictors influencing cervical cancer screening uptake among women aged 30‐49 years in sub-Saharan Africa.

**Methods:**

For this study, a weighted dataset of 33,952 individuals from the 2022 Demographic and Health Survey in Ghana, Kenya, Mozambique, and Tanzania was used. STATA version 17 (StataCorp) and Python 3.10 (Python Software Foundation) were used for data preprocessing and analysis. MinMax and standard scaler were applied for feature scaling, and recursive feature elimination was used for feature selection. An 80:20 ratio was applied for data splitting. Tomek links with random oversampling were used for handling class imbalance. A total of 7 models were selected and trained using both balanced and unbalanced datasets. Model evaluation was performed using area under the receiver operating characteristic curve, accuracy, and a confusion matrix.

**Results:**

The proportion of cervical cancer screening in sub-Saharan Africa was 13%, which is lower than reported in previous studies. Random forest was the best-performing model, achieving an accuracy of 78%, an area under the curve of 86%, an *F*_1_-score of 79%, a recall of 81%, and a precision of 77%. The waterfall plot’s Shapley Additive Explanations analysis showed that wealth status, awareness of sexually transmitted infections, HIV testing exposure, age at first sexual intercourse, educational level, residency, smartphone ownership, having a single sexual partner, and previous health status were predictors of cervical cancer screening.

**Conclusions:**

Improving education and awareness, expanding access to screening (especially in rural areas), leveraging both digital health and community-based outreach, integrating screening with other health services, and addressing socioeconomic barriers are recommended strategies to increase cervical cancer screening rates in sub-Saharan Africa.

## Introduction

Cervical cancer has been ranked as the fourth most common cancer affecting women, contributing to approximately 660,000 new diagnoses and 350,000 fatalities globally [1]. About 75%‐85% of these cases occur in low-income countries, with 23% specifically in Africa (as reported by the World Health Organization [WHO]). The incidence and mortality rates of cervical cancer are 2-3 times higher in low-income countries compared to high-income countries [2].

Cervical cancer ranks as the second leading cause of cancer-related death globally [3]. In 2025, studies from Africa showed that cervical cancer screening prevalence differs significantly between high-income countries (84%) and low- and middle-income countries (15%) [4]. Also, in Africa, more than 53% of cervical cancer cases are identified at an advanced stage (stage III-IV) [5].

Cervical cancer is “a malignant tumor that forms when the cells in the tissue surrounding the cervix grow and multiply uncontrollably by passing the normal process of cell division” [6]. Effective early screening has been shown to reduce cervical cancer incidence by up to 80% and prevent more than 40% of new cases [2,7].

With an average cure rate of 80%, radiation therapy effectively treats cervical cancer in its early stages [8]. However, the practice of early screening, before the disease reaches an advanced stage, continues to be a major problem in many parts of Africa [5].

Despite the implementation of screening initiatives and the introduction of the human papillomavirus (HPV) vaccine, cervical cancer remains a significant public health concern in Africa [9].

Early detection and timely treatment of cervical cancer are crucial [10]. The HPV has more than 100 serotypes, but the HPV vaccine targets only 9 of them [11]. Also, early screening is a key approach to achieving 1 of the 3 WHO 90-70-90 pillars: “90% of girls fully vaccinated against HPV, 70% of women screened by age 35 and 45 with a high-performance test, and 90% of women with precancerous lesions receiving treatment” [12,12].

Therefore, early screening is essential to enhance the probability of early diagnosis and treatment. Early screening is guaranteed to identify precancerous lesions and cancer before symptoms appear, which is the goal of screening, and enhances the chances of successful treatment and reduces maternal mortality [12-14].

However, studies show that screening in Africa is conducted at less than 70% of the WHO target [15]. This is due to a perceived lack of expertise, limited knowledge about cervical cancer, inadequate screening tools and supplies, and insufficient funding [5,16,17]. In the recent study, the prevalence of precancerous cervical lesions and cancer is relatively high, at 18% and 14%, respectively [18].

According to previous studies conducted in Africa, age, education level, employment status, wealth index, place of residence, and access to health care facilities were common predictors of cervical cancer screening among women of reproductive age [19-22].

Although studies have been conducted in African countries, they were limited to small sample sizes and single countries, lacking representativeness. This study, however, is representative, with a large dataset covering multiple countries, providing reliable evidence for policymakers and researchers to inform interventions.

The health care system produces vast amounts of data that must be analyzed to support effective decision-making. As the volume and complexity of this data grow, relying on traditional analytical methods without the aid of machine learning is becoming increasingly impractical.

A key strength of machine learning is its ability to handle big datasets while delivering reliable performance metrics. Although this study focuses on sub-Saharan Africa, the application of machine learning in this context allows for the extraction of meaningful patterns from complex health data.

## Methods

### Data Processing and Management

**Figure 1.**
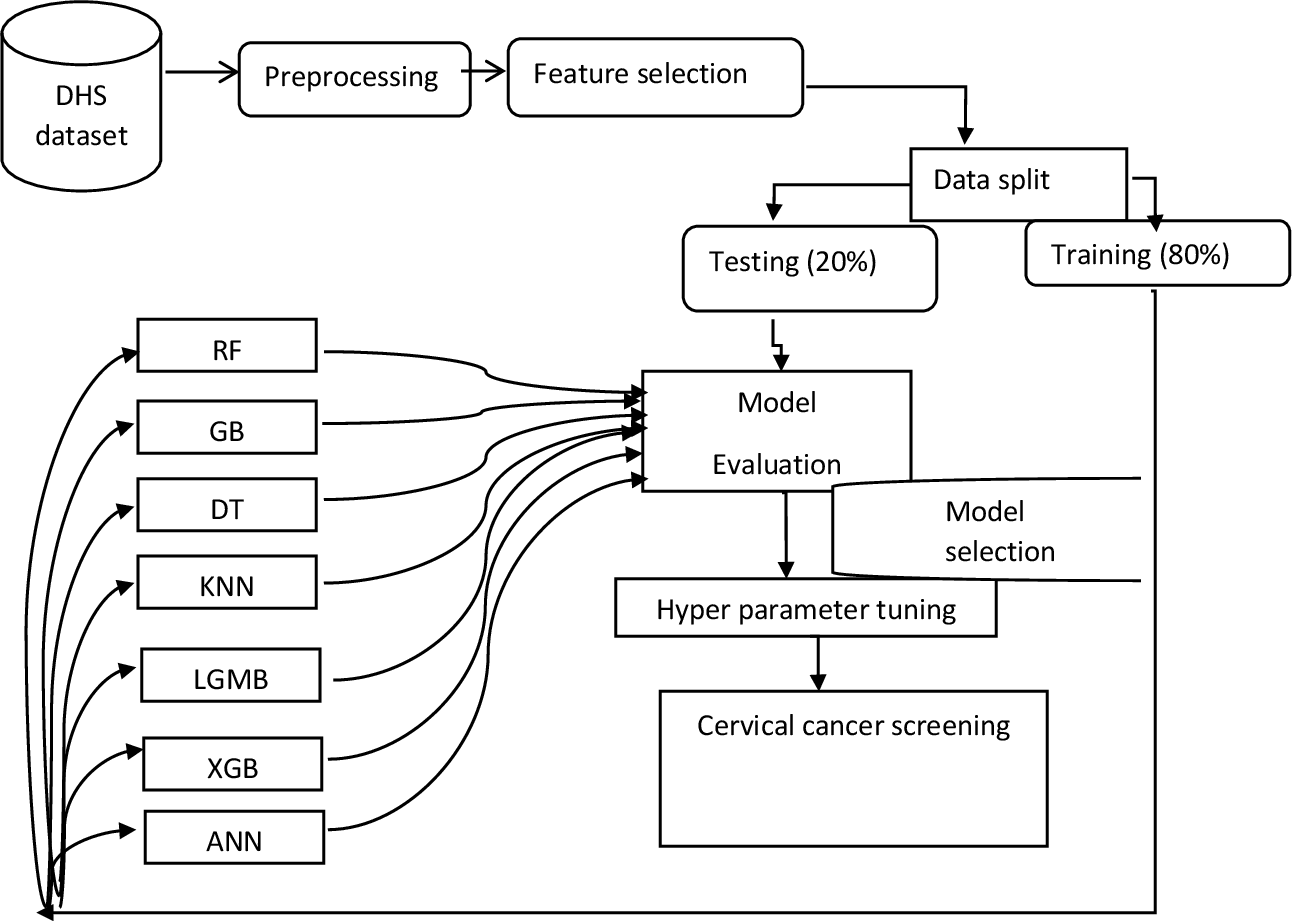
Overview flowchart of data preparation and analysis plan applied. ANN: artificial neural network; DHS: Demographic and Health Surveys; DT: decision tree; GB: gradient boosting; KNN: K-nearest neighbors; LGMB: light gradient boosting machine; RF: random forest; XGB: extreme gradient boosting.

After deciding on the title by considering the current public health issue, data were extracted from the individual record dataset. Then, the relationships between all variables and the outcome were reviewed based on previous study evidence and expert opinions. The similarity of the selected variables across 4 countries (Ghana, Kenya, Mozambique, and Tanzania) that had recent (2022‐2023) Demographic and Health Surveys (DHS) data available was checked. STATA version 17 (StataCorp) and Python 3.10 (Python Software Foundation) were used for data processing and management. To handle missing data, mode imputation and K-nearest neighbors imputation were used to maintain the data distribution, ensure statistical consistency, and preserve the integrity of the dataset (Figure 1).

Many machine learning algorithms are sensitive to the scale of features and need normalization before modeling [23]. MinMaxScaler and StandardScaler were applied to normalize or standardize our data, ensuring a consistent scale and improving the performance of modeling tasks. The goal is to minimize performance bias in models that are sensitive to feature scaling, as the absence of standardization can negatively impact model performance.

### Feature Selection

**Figure 2.**
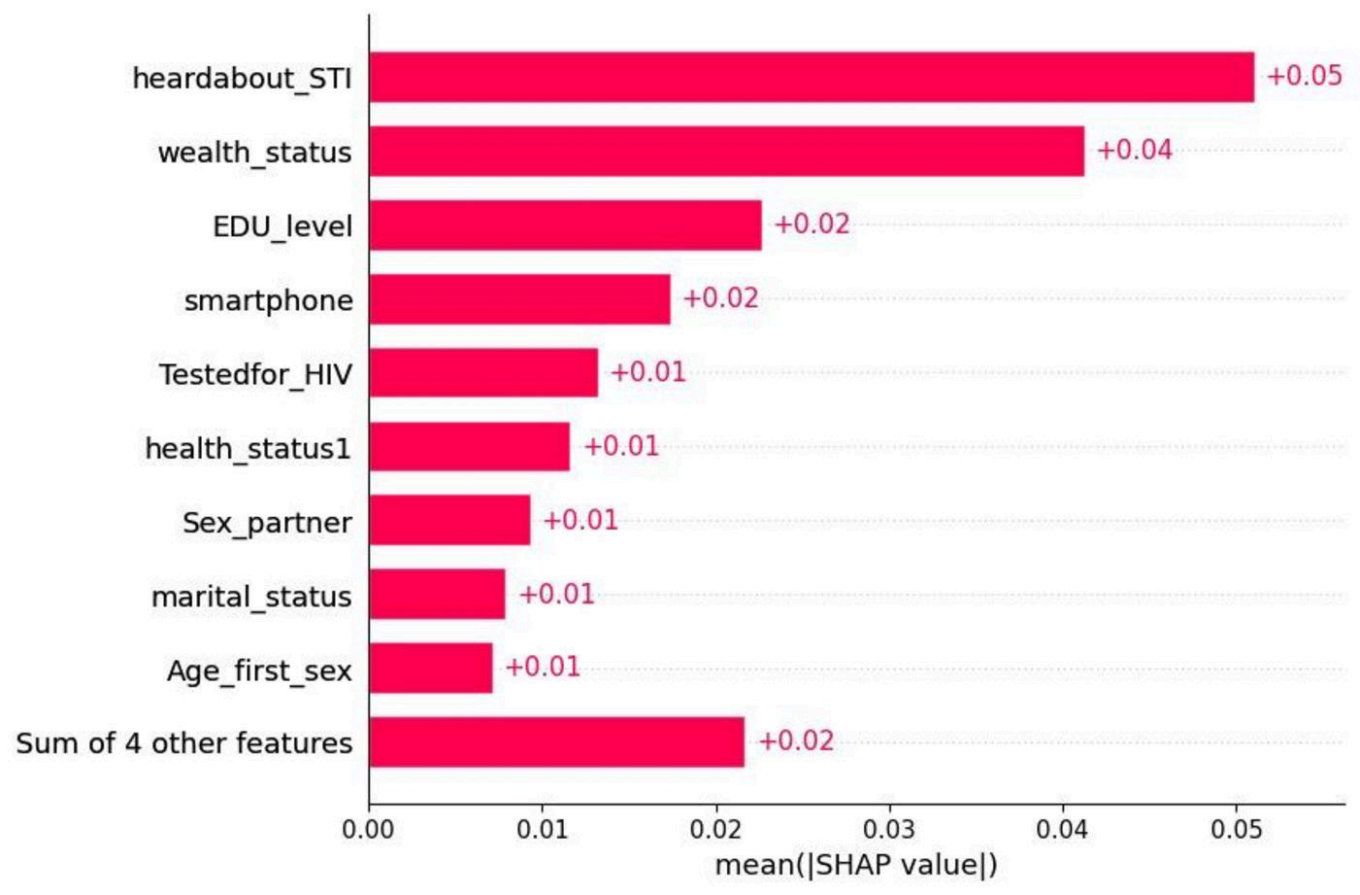
The top 10 features of cervical cancer screening. SHAP: Shapley Additive Explanations.

Recent studies indicate that irrelevant variables weaken the model’s capacity for generalization, raise its overall complexity, and possibly lower a classifier’s overall accuracy in a machine learning study [24]. Recursive feature elimination was used to methodically remove irrelevant features, decreasing dimensionality while retaining the most important predictive variables, thereby enhancing the model’s efficiency and ability to generalize (Figure 2).

### Splitting the Data

Previous studies indicate that data splitting offers a trustworthy estimation of the model’s performance on unseen data and aids in the detection of overfitting [25]. For data splitting, an 80:20 ratio was used for training and testing, respectively, to assess the model’s performance and prevent overfitting in machine learning algorithms.

### Handling Imbalanced Data

In machine learning, class imbalance is frequently encountered due to unbalanced data, and this can have a big influence on model accuracy [26].

To improve model generalization and minimize noise and class overlap, we applied the synthetic minority oversampling technique (SMOTE) in combination with Tomek links undersampling. This process resulted in a balanced and cleaner dataset, where the original distribution of 3878 “yes” cases and 30,074 “no” cases was adjusted to 30,074 instances in each class.

### Model Selection

After a detailed review of machine learning studies conducted on reproductive health, 7 models were selected based on simplicity, accuracy, robustness, and computational efficiency, including decision tree, random forest (RF), K-nearest neighbors, extreme gradient boosting, light gradient boosting machine, adaptive boosting, and gradient boosting (GB).

### Model Training

Both balanced and unbalanced datasets were used to train the selected classifiers after the model was selected. They used 10-fold cross-validation to assess their performance. In order to generate final predictions on test data that had not yet been observed, the best successful predictive model was selected after the comparison and trained using balanced training data.

### Hyperparameter Tuning

A crucial step in maximizing the potential of machine learning models and ensuring their accuracy, efficiency, and robustness is hyperparameter tuning. Hyperparameter tuning by RF is listed in Table 1.

**Table 1.** Hyperparameters and values for all models.

Classifier	Hyperparameter and value
DT^a^	max_depth=10, criterion=gini, min_samples_split=10, min_sample_leaf=1,
RF^b^	max_depth=20,min_samples_split=10, n_estimators=300
KNN^c^	n_neighbors=5, weights='uniform’, metric='minkowski’
ANN^d^	hidden_layer_sizes=(100,), activation='relu’, solver='adam’, learning_rate='adaptive’, max_iter=300
XGB^e^	colsample_bytree=0.8, learning_rate=0.05, max_depth=3, n_estimators=100, subsample=1.0
LGBM^f^	learning_rate=0.1, n_estimators=20, num_leaves=31
ADA^g^	n_estimators=300, learning_rate=1.0
GB^h^	n_estimators=300, learning_rate=0.1, max_depth=3, min_samples_split=10

a DT: decision tree.

b RF: random forest.

c KNN: K-nearest neighbors.

d ANN: artificial neural network.

e XGB: extreme gradient boosting.

f LGBM: light gradient boosting machine.

g ADA: adaptive boosting.

h GB: gradient boosting.

### Model Evaluation

**Figure 3.**
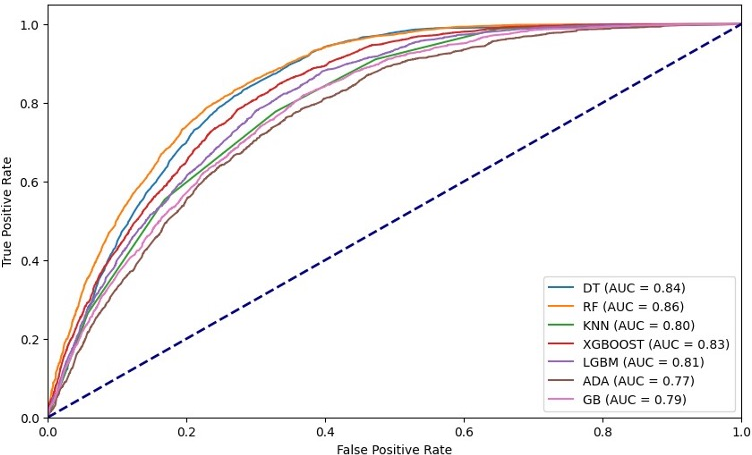
ROC curve for model comparison to predict cervical cancer screening. ADA: adaptive boosting; AUC: area under the curve; DT: decision tree; GB: gradient boosting; KNN: K-nearest neighbors; LGMB: light gradient boosting machine; RF: random forest; ROC: receiver operating characteristic; XGB: extreme gradient boosting.

Model evaluation was performed using confusion matrix, *F*_1_-score, area under the receiver operating characteristic curve, accuracy, precision, and recall in this study. Ultimately, the RF model outperformed others due to its effectiveness in handling nonlinear relationships, evaluating feature importance, and minimizing overfitting through the ensemble of multiple decision trees. For results, see Figure 3 and Tables2  3.

**Table 2.** Model evaluation after synthetic minority oversampling technique.

Model	Accuracy (%)	AUC^a^ (%)	*F*_1_-score (%)	Recall (%)	Precision (%)
DT^b^	78	85	78	80	76
RF^c^	78	86	79	81	77
KNN^d^	72	80	74	75	73
XGBoost^e^	76	83	76	78	75
LGBM^f^	74	81	75	76	74
ADA^g^	72	77	71	73	69
GB^h^	72	79	72	74	70

a AUC: area under the curve.

b DT: decision tree.

c RF: random forest.

d KNN: K-nearest neighbors.

e XGBoost: extreme gradient boosting.

f LGBM: light gradient boosting machine.

g ADA: adaptive boosting.

h GB: gradient boosting.

**Table 3.** Confusion matrix for cervical cancer prediction by random forest accuracy (2802+2494+837+658=6791=78%).

	Predicted positive	Predicted negative
Actual positive	2802 (true positive)	658 (false negative)
Actual negative	837 (false positive)	2494 (true negative)

### Ethical Considerations

This study did not require institutional review board approval because it involved secondary analysis of publicly available and deidentified data from the DHS Program. As a result, a consent letter for data access was obtained from a major health and demographic survey program via a web-based request submitted at the DHS Program website [27]. All data used in this study consisted of nonidentifiable information.

## Results

### Cervical Cancer Screening Distribution by Country

The proportion of women screening rates in Kenya (14%), Mozambique (13%), Tanzania (12%), and Ghana (7%) remains low (Table 4).

**Table 4.** Cervical cancer screening distribution by country.

Country	Categories	
	Yes, n (%)	No, n (%)
Ghana	530.8 (7)	6767.2 (92)
Kenya	2053 (14)	12,389 (85)
Mozambique	669 (13)	4575.8 (88)
Tanzania	842.6 (12)	6068.1 (88)

### Individual Characteristics of Cervical Cancer Screening

The majority of women living in rural (17,987.6/19,690.1, 91%) and urban areas (11,812.7/14,205.8, 83%) have not undergone cervical cancer screening. Among women who own a smartphone, 2170.8 of 11,628.9 (19%) have been screened for cervical cancer, while only 1924.8 of 22,267.0 (9%) of those without a smartphone have been screened.

Women who self-report good health status have a cervical cancer screening rate of 15% (156.9/1,056.6) compared to 11% (2762.9/24,302.6) among those who report poor health status. Women with high wealth status have a cervical cancer screening rate of 18% (2884.8/15,923.9), compared to 5% (604.5/11,398.8) among those in the poorest wealth category.

Only 286.1 of 1875.2 (15%) women who use family planning methods have been screened for cervical cancer. Those who do not consider distance a big problem had a slightly higher screening rate (3226.1/26,048.9, 12%). Women who initiated sexual activity after the age of 30 years had a screening rate of 14% (1932.9/13,527.5), higher than the 11% (2162.7/20,368.2) among those who began before the age of 30 years.

Women reporting multiple sexual partners had a significantly higher screening rate (793.2/4218, 19%) than those who did not (3302.5/29,677.9, 11%). Women who have been tested for HIV have a cervical cancer screening rate of 13% (3958.3/29,777), compared to only 3% (137.3/4118.9) among those who have not been tested. Divorced and widowed women show slightly higher screening rates at 15% (676.3/4638.9) and 14% (258.5/1879.7), respectively.

Women with tertiary education have a screening rate of 22% (838.7/3738.8), compared to only 4% (238.6/6572.2) among those with no education. Women who have heard about sexually transmitted infections (STIs) have a screening rate of 17% (3900.8/23,313.8), compared to only 1.9% (194.8/10,582.1) among those who have not (Table 5).

**Table 5.** Individual characteristics of cervical cancer screening.

Variables and categories		Yes, n (%)	No, n (%)
Residence			
Urban		2393.1 (17)	11,812.7 (83)
Rural		1702.5 (9)	17,987.6 (91)
Frequently social media usage			
No		2873.5 (12)	20,629.5 (88)
Yes		1222.2 (12)	9170.8 (88)
Availability of smartphone			
No		1924.8 (9)	20,342.2 (91)
Yes		2170.8 (19)	9458.1 (81)
Self-report of health status			
Good		156.9 (15)	899.7 (85)
Moderate		1175.9 (14)	7360.8 (86)
Poor		2762.9 (11)	21,539.7 (88)
Wealth			
Lowest		604.5 (5)	10,794.3 (94)
Middle		606.3 (9)	5966.9 (91)
Highest		2884.8 (18)	13,039.1 (82)
Family planning method use			
No		3809.5 (12)	28,211.2 (88)
Yes		286.1 (15)	1589.1 (84)
Distance to health facility			
Big problem		869.6 (11)	6977.4 (88)
Not a big problem		3226.1 (12)	22,822.8 (87)
Age at first sexual intercourse (years)			
>30		1932.9 (14)	11,594.6 (85)
<30		2162.7 (11)	18,205.5 (89)
Have multiple sexual partners			
No		3302.5 (11)	26,375.4 (89)
Yes		793.2 (19)	3424.8 (81)
Tested for HIV/AIDS			
No		137.3 (3)	3981.6 (96)
Yes		3958.3 (13)	25,818.7 (86)
Marital status			
Single		254 (13)	1751.7 (87)
Married		2906.7 (12)	22,464.6 (89)
Widowed		258.5 (14)	1621.2 (86)
Divorced		676.3 (15)	3962.6 (85)
Educational status			
No education		238.6 (4)	6333.6 (96)
Primary		1668.7 (12)	12,746.3 (88)
Secondary		1349.6 (15)	7820.2 (85)
Tertiary		838.7 (22)	2900.1 (78)
Heard about STI^a^			
No		194.8 (1.9)	10,387.3 (98)
Yes		3900.8 (17)	19,413 (83)

a STI: sexually transmitted infection.

### Determinants of Cervical Cancer Screening

In machine learning, Shapley Additive Explanations (SHAP) values indicate how each feature impacts the output variable. This study examines the impact of the top 10 features on women’s cervical cancer screening status. These insights are crucial for specific interventions and policy formulation aimed at enhancing cervical cancer screening practices, as well as reducing morbidity and mortality among women.

**Figure 4.**
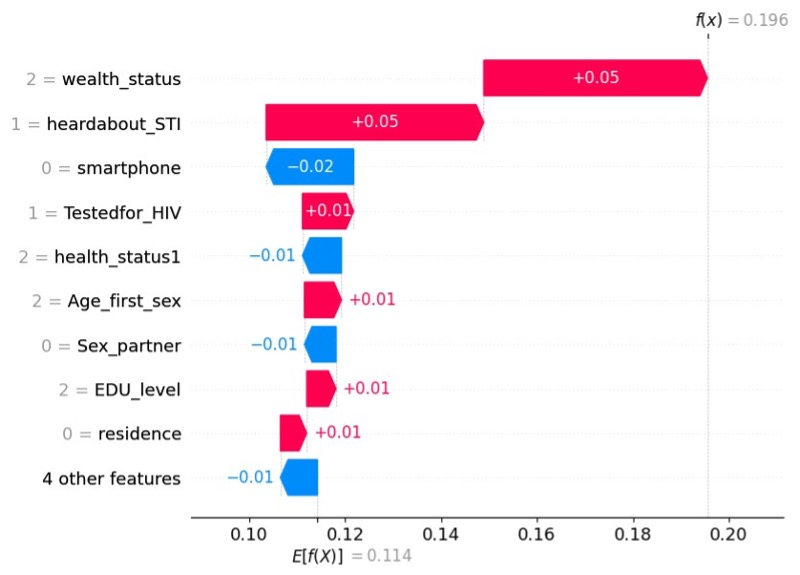
Determinants of cervical cancer screening visualized by a waterfall plot.

In Figure 4, a feature contributing positively to the predicted screening practice is represented by a positive SHAP value (red), while a feature contributing negatively to the predicted screening outcome is represented by a negative SHAP value (blue).

The best predictive model (RF) showed that wealth status, awareness of STIs, HIV testing, age at first sex, primary education and above, and living in urban areas are significant factors associated with increased cervical cancer screening. However, factors such as not owning a smartphone, having a single sexual partner, and unknown health status are associated with a decrease in cervical cancer screening (Figure 4).

## Discussion

### Principal Findings

In sub-Saharan Africa, cervical cancer is the second most common cause of cancer-related death on the continent and the fourth leading cause of cancer-related death globally [28,29]. Low- and middle-income countries account for nearly 85% of cervical cancer cases and related deaths [28]. However, according to this study, a small percentage of women were screened for cervical cancer in Kenya (14.22%), Mozambique (12.76%), Tanzania (12.20%), and Ghana (7.27%). This low screening rate contributes to delays in early cancer management and highlights the urgent need for targeted interventions to improve screening behaviors among women.

The predictive evidence from this machine is used to support the realization of the 2030 plan [30]: 90% of cervical diseases identified, 90% of precancerous cases treated, and 90% of women with invasive cancer managed.

The RF is the best-performing model, achieving an accuracy of 78%, an area under the curve of 86%, an *F*_1_-score of 79%, a recall of 81%, and a precision of 77%. The waterfall plot’s SHAP analysis indicates that variables, such as middle and higher wealth status, awareness of STIs, HIV testing, age at first sex, primary education and above, and living in urban areas are significant factors associated with increased cervical cancer screening.

However, not having a smartphone, having a single sexual partner, and being unaware of health status are associated with a decrease in cervical cancer screening.

In this study, the proportion of cervical cancer screening is 13%, which is higher than findings from studies conducted in Cameroon (4%) [2], South India (7.1%) [31], and lower than those reported in Ethiopia (14.79%) [32], and sub-Saharan Africa (19%) [33]. Possible justifications for this difference may include public awareness levels, accessibility of screening services, outreach programs, community health education, socioeconomic status, and geographic barriers. Differences in study populations, sampling methods, and time frames may also contribute to these variations [34].

According to this finding, women with middle and higher wealth status have increased cervical cancer screening rates, which is supported by previous research conducted in the United States, Ethiopia, Uganda, and Cameroon [2,35-37]. One explanation could be that women with middle-class and upper-class incomes have easier access to health care because they can afford the direct and indirect costs of medical care, including transportation and other costs.

This finding shows that women who have information about STIs are more likely to undergo cervical cancer screening. This is supported by previous studies conducted in Southern Ethiopia, Eastern and Southern Africa, Haiti, and Morocco [38-41]. This is because women who have information about STIs are also exposed to information about cervical cancer, including the benefits of early screening, and they tend to have better health-seeking behavior.

According to this SHAP finding, women who do not have smartphones show decreased cervical cancer screening rates. This is supported by previous studies conducted in Japan and England [42,43]. Those who have smartphones can easily access health information, including preventive health care such as screenings, through websites, mobile apps, and social media. Those who do not have smartphones may miss this information and, as a result, may not pay attention to screening [44].

The SHAP finding showed that women who have previously been tested for HIV are more likely to undergo cervical cancer screening. This is consistent with previous studies conducted in Uganda, China, Kenya, and Ethiopia [45-48].

Also, this study indicated that women who did not have information about their health status had decreased cervical cancer screening practices. This is supported by previous studies conducted in Nigeria, Ghana, Australia, India, and Kenya [49-53]. Women who do not have information about their health status are less likely to undergo cervical cancer screening because a lack of awareness reduces their understanding of risk, engagement with health care, and motivation to seek preventive services.

This study showed that women who had their first sexual intercourse before the age of 30 years had an increased cervical screening status. This is in line with studies conducted in East Gojjam Zone, Ethiopia, Africa, and Nigeria [54-56]. A possible explanation for this is that early sexual activity is associated with a higher likelihood of STIs, which in turn leads to more frequent visits to health facilities due to increased awareness of cervical cancer risk.

In addition, women in this age group are more educated and more likely to understand messages from social media and health professionals, including information about cervical cancer risk and the benefits of early screening and treatment [56].

SHAP findings showed that women who have a single sexual partner have decreased cervical cancer screening rates. This finding was supported by studies conducted in Korea, Jordan, Ethiopia, and Uganda [57-60].

Women with a single partner have high confidence that they are free from cervical cancer risk factors because, during health facility visits and through social media information, they understand that women with single sexual partners are at a lower risk of contracting HPV and other STIs, which are the main causes of cervical cancer [61].

This finding also showed that women with primary and higher education levels have increased cervical cancer screening status, which is in line with previous studies conducted in Romania, the Southwest United States, Mayuge, Ghana, and Korea [62-66].

A possible explanation for this could be that educated women have greater health literacy, allowing them to easily understand basic health information and services, including information about cervical cancer. Educated women also tend to have better access to resources such as health care providers, smartphones, and official websites like those of the WHO.

In addition, they may be less influenced by cultural misconceptions or stigma surrounding cervical cancer screening [67].

SHAP findings also showed that women residing in urban areas had increased cervical cancer screening rates. This is in line with previous studies conducted in Nepal, Nigeria, China, and Ghana [55,68-70]. Women residing in urban areas have improved access to health information and services through social media, public health campaigns, and proximity to health care facilities and professionals. Consequently, they tend to have a better understanding of cervical cancer compared to their rural counterparts [71].

### Strengths and Limitations

One of the study’s strengths is that it makes use of a large and varied dataset, which improves the findings’ applicability to a larger geographic area. Furthermore, complex patterns and relationships that traditional statistics can miss might be found through the use of machine learning models, and the limitations of this include issues that can impact prediction accuracy, such as self-reported information, recall bias, and missing data. Furthermore, it is challenging to determine causal links due to the cross-sectional nature of data, and the findings based on machine learning outputs should be interpreted as associations rather than causations.

### Conclusions

Wealth status, awareness of STIs, HIV testing exposure, age at first sexual intercourse, educational level, residency, smartphone ownership, having a single sexual partner, and previous health status are predictors of cervical cancer screening.

Improving education and awareness, expanding access to screening, especially in rural areas, leveraging both digital health and community-based outreach, integrating screening with other health services, and addressing socioeconomic barriers are recommended strategies to increase cervical cancer screening rates in sub-Saharan Africa.

## Abbreviations

DHS: Demographic and Health Survey
LR: logistic regression
RF: random forest
SHAP: Shapley Additive Explanations
STI: sexually transmitted infection
WHO: World Health Organization
